# Experiences and attitudes of residents regarding a community-based genome cohort study in Japan: a population-based, cross-sectional study

**DOI:** 10.1186/s12920-016-0175-8

**Published:** 2016-03-15

**Authors:** Keiko Miyamoto, Miho Iwakuma, Takeo Nakayama

**Affiliations:** Department of Medical Communication, Kyoto University School of Public Health, Yoshidakonoe-cho, Sakyo-ku, Kyoto #6068501 Japan; Department of Health Infomatics, Kyoto University School of Public Health, Yoshidakonoe-cho, Sakyo-ku, Kyoto #6068501 Japan

**Keywords:** Public attitude, Genome study, Drug susceptibility, Familiarity, Japan

## Abstract

**Background:**

Because of the rapid development in genomics, more research findings have emerged. However, the association between society and research results remains controversial. This article examines the experiences and attitudes of residents regarding a community-based genomic cohort study.

**Methods:**

This study was conducted as a part of the health survey of the City Health Promotion section. At the conclusion of the first stage of the project, a self-administered questionnaire was mailed to a random sample of 2,500 residents in 2012.

**Results:**

The response rate was 59 % (*n* = 1477/2500). The findings show that 70 % of males and 50 % of females knew nothing about the project. Females and elderly people were more likely to have knowledge of the study, indicating that self-rated understanding of the terminology is statistically associated with the level of awareness regarding the project. In addition, those who were aware of the project were also aware of the benefits of research utilizing genetic information, whereas unaware respondents, particularly males, believed that unexpected negative effects may occur. Those with higher self-rated understanding of the terminology and higher awareness of benefit of the research utilizing genetic information had more positive attitudes toward undergoing drug susceptibility genetic testing, indicating that the awareness of project in females and concerns toward genetic research are not statistically associated with the willingness to undergo.

**Conclusions:**

This study suggests that a community-based genome cohort project helps raise awareness of benefit of genetic research and that knowledge, however, does not directly affect the willingness to participate in related activities, such as drug susceptibility genetic testing. Therefore, additional research that focuses on the circular relationship between risk and action must be conducted in the future.

## Background

Since the human genome was sequenced in 2003, the field of genome epidemiology has produced numerous studies on the complex relationships between phenotypes and genotypes [1]. The focus has shifted away from medical institutions to communities, which has created a greater need for members of the general public to provide sensitive information regarding their genetics, lifestyle habits, and medical histories depending on trust [2, 3]. Therefore, public attitudes toward genome research can significantly affect willingness to support such studies.

The increase in genomic studies has also shown the difficulty of applying study outcomes to clinical practice [4–6]. Direct-to-consumer genetic testing that bypasses medical settings [7, 8] has also become controversial. In addition, because of accumulating evidence that drug susceptibility can differ depending on genotypes, there are persistent expectancies for personalized medicine based on pharmacogenomics [9–11]. Better understanding of public attitudes and awareness toward genome studies could thus lead to improved integration of genome research into medical plans and procedures [12, 13].

In 2005, the Kyoto University Graduate School of Medicine and the city of Nagahama initiated the Nagahama Study for Comprehensive Human Bioscience (hereafter referred as the Nagahama study) to promote the health of Nagahama citizens, develop community-based genomic-epidemiologic studies, and create a biobank. In particular, the Nagahama study examined the effects of the complex gene–environment interaction on diseases by storing biological samples of Nagahama residents (aged 30–74 years) and following their conditions for 10 years. The main study began in November 2008. From November 2006 to November 2010, the Kyoto University researchers held 13 public forums on contemporary medical issues, such as the genome, lifestyle-related diseases, sleep disorders, information disclosure, and hospital evaluations for residents or high school students. These meetings included six symposia and five “science café” talks; these events are ongoing. For recruitment, the Nagahama City Health Promotion section provided leaflets through resident associations and placed announcements in Nagahama-area media regarding the study program. In addition, residents aged 30–39 were contacted by mail, while those aged 40–74 were asked to serve as an alternative of the national health check-up program in Japan. Ultimately, 10,084 citizens (14 % of the eligible people) participated in the Nagahama study program. According to the findings of a 2009 midterm study on citizens’ awareness of and attitudes toward the program, the most commonly cited positive aspect of the Nagahama study was the “free and extensive health check-up program.” Half of those who had participated in Nagahama study knew the Nagahama study was a genome study [14].

There are several quantitative studies regarding public attitudes toward genome studies [15–19]. They suggest that the majority of lay people have positive attitudes toward genome studies. In some studies, positive attitudes toward genome studies were associated with genome literacy [16, 17]. However, because the respondents’ attitudes were changeable depending on the contexts, the authors concluded that negative attitudes do not simply reflect a lack of understanding [15, 20, 21]. Sturgis & Allum [22] suggested that the knowledge of science engenders positive attitudes toward science. However, it may be useful to integrate knowledge and context to understand the public attitudes toward science because science is not isolated in society.

Another background characteristic associated with positive attitudes toward genome study is familiarity or the experience of genetic study and awareness of terminology [15, 17]. The Dutch study by Henneman et al. [16] clarified that respondents who have heard of genetic testing anticipated greater importance of the genetic aspects of diseases. They suggested that those who were familiar with a genetic disease were more likely to support the use of genetic tests and that the respondents’ attitudes were not always associated with the level of genetic knowledge.

Some studies have focused on the association between familiarity and public attitudes toward nanotechnology [23–25], while toward genetic testing are limited. Henneman et al. [26] conducted a survey in 2010 (in addition to 2002) about public attitudes toward genome studies and investigated the change in attitudes over the preceding 8 years. Their results revealed that people were more interested in their own genetic makeup, but experience in genetic testing had not changed. In Nagahama, Miyamoto et al. [14] clarified that the Nagahama study was accepted by the general public, who were somewhat disconnected from the world of science. Therefore, by focusing on their experiences, this study examines how a community-based genomic–epidemiologic study affected the residents’ attitudes toward genome studies. In addition, the association between residents’ experiences and willingness to participate in drug susceptibility genetic testing was explored.

## Method

### Participants and setting

This study was conducted as a part of the health and lifestyle survey of the Nagahama City Health Promotion section to examine the health consciousness of the residents of Nagahama. Data were collected using an anonymous self-administered postal questionnaire in March and April 2012. The return of the questionnaire was viewed as implied consent. In compliance with local regulations, a random sample of 2,500 subjects (aged 30–69 years) was selected from the Basic Resident Register. The study protocol was approved by the Kyoto University Graduate School and Faculty of Medicine Ethics Committee.

### Measures

The questionnaire was developed on the basis of preliminary surveys conducted during earlier phases of the Nagahama study [14, 27] and the nationwide survey on public attitudes toward genetic studies in Japan [17]. Revisions were made according to advice from public health researchers and staff members of the Nagahama City Health Promotion section. The survey items are listed below.

### Attitudes toward the use of genetic information for medical purposes

The three “awareness of benefit” items were “helpful for disease diagnosis,” “helpful for disease treatment,” and “helpful for disease prevention.” The five “concern” items were “studies require financial infusion,” “privacy concerns are raised,” “discrimination in employment and when purchasing insurance is generated,” “cloned human beings come into existence,” and “unexpected negative effects may be raised” and one “belief” item was “companies or government bodies use genome information.” The answers were scored on a five-point Likert scale ranging from 1 (completely disagree) to 5 (completely agree).

### Willingness to participate in drug susceptibility genetic testing

On the basis of a hypothetical scenario, **t**he following was included in the survey: “Hypothetically speaking, there is a drug that has a good effect on some, whereas it has an adverse effect on others. When you undergo drug susceptibility genetic testing, you are informed of the drug’s effectiveness or adverse effects. Would you still donate your DNA for the test?” The answer was scored on a five-point Likert scale ranging from 1 (never) to 5 (definitely yes).

### Individual factors

In addition to the survey items, each participant was asked to provide socio-demographic characteristics (age, sex, employment status, and formal education duration). The latter question’s responses were dichotomized to less than 12 years (high school graduate and lower) or more than 13 years (college and higher). Participants were also surveyed as to the level of awareness regarding the three aspects of the Nagahama study (the extensive health check-up program, follow-up study, and genome studies). The awareness of the Nagahama study was dichotomized between “High awareness of the Nagahama study (those who knew more than one content)” and “Low awareness of the Nagahama study (those who knew nothing about the study’s content). Furthermore, the participants were surveyed on the self-rated understanding of the terms “genome” and “gene.” Answers to the questions consisted of the following: “I understand it,” “I have heard of it,” and “I have never known about it.” Then, the respondents were divided into three groups: “High” knowledge level was defined as understanding both terms or understanding “gene” and having heard of “genome;” “middle” knowledge was defined as having heard of both of terms or understanding “gene” and having never known about “genome;” and “low” knowledge was defined as having heard of “gene” and not “genome” or having never known about both the terms.

### Analyses

All data analyses were performed using SPSS version 22.0 J for Microsoft Windows. Chi-square tests were used to examine associations between the items. The difference was considered significant at *p* < 0.05 (two-sided test). Logistic regression analyses were employed to identify factors associated with the awareness of benefits of genome study and the willingness to undergo the drug susceptibility genetic testing as dependent variables, respectively. Both dependent variables were dichotomized between those who agreed with the questions versus neutral/disagree.

## Results

### Respondents’ characteristics

Out of the 2,500 questionnaires distributed, 1,477 were returned (59.1 % response rate). Two blanks and two of unknown sex were excluded for a total of 1,473. Demographic and individual factors including awareness of the research contents of the Nagahama study and self-rated understanding of the terminology are presented in Table 1. Overall, males (47.8 %) in their 30s (18.5 %) and 40s (21.5 %) and those having completed less than 12 years of formal education (57.6 %) were underrepresented.

**Table 1:** Demographic awareness of the Nagahama study, self-rate understanding of terminology, and willingness toward drug susceptibility genetic testing

	Male (*n* = 704)		Female (*n* = 769)	
	%	n	%	n
Age group (years)				
30–39	16.1	112	20.7	159
40–49	20.1	140	22.8	175
50–59	23.8	166	26.3	202
60–69	40.0	279	30.2	232
Formal educational period				
High school graduate and lower	59.1	392	56.3	414
College and higher	40.9	271	43.8	322
Awareness of the Nagahama study contents				
Know more than one content	30.6	210	50.3	381
Know nothing	69.7	483	49.7	376
Self-rated understanding of terminology				
High^a^	35.7	251	24.1	185
Medium^b^	35.1	247	35.0	269
Low^c^	28.0	197	38.4	295
Willingness toward drug susceptibility genetic testing (Would you like to donate your DNA for drug susceptibility genetic testing?)				
Yes	49.3	339	47.9	360
Neutral	27.2	187	32.0	240
No	23.4	161	20.1	151

^a^Understanding “gene” and “genome” and understanding “gene” and having heard of “genome”

^b^Having heard of “gene” and “genome” or understanding “gene” and having never known about “genome”

^c^Having of “gene” and not “genome” or having never known about “gene” nor “genome”

As for the awareness of project’s contents, respondents were asked whether they knew about the three aspects of the Nagahama study. According to the results, 9.8 % of the males and 14.5 % of the females knew about all three items; 20.5 % of the males and 35.8 % of the females knew about one or two items; and 69.7 % of the males and 49.7 % of the females knew none of the three. With regard to self-rated understanding of the terms “genome” and “gene,” “High” knowledge level as understanding both terms (*n* = 159) or understanding “gene” and having heard of “genome” (*n* = 277) was 30.2 %; the “middle” level as having heard of both of terms (*n* = 377) or understanding “gene” and having never known about “genome” (*n* = 139) was 35.7 %; and “low” knowledge consisted of having heard of “gene” and not “genome” (*n* = 472) or having never known about both terms (*n* = 20) was 34.1 %. Males were more prevalent in the high level than females [*χ*^2^ (2) = 28.47, *p* < 0.001].

Respondents who knew about all or some of the items (*n* = 591) were 40.7 % and those who knew nothing (*n* = 859) were 59.3 %. Males and younger people were less aware of the Nagahama study [male, *χ*^2^ (1) = 60.10, *p* < 0.001; younger, *χ*^2^ (3) = 19.90, *p* < 0.001]. There was no significant difference in educational status regarding awareness of the Nagahama study [*χ*^2^ (1) = 2.26, *p* = 0.133]. Awareness of the Nagahama study predicted a significantly higher level of understanding of the terms “gene” and “genome” [*χ*^2^ (2) = 20.77, *p* < 0.001] (Table 2).

**Table 2:** Differences in individual factors between those who were aware of the Nagahama study and those who knew nothing about the Nagahama study from Qui-square test

	Male (*n*=704)					Female (*n*=769)				
	Aware of the Nagahama study (*n*=210) %	Know nothing (*n*=483) %	OR	95 % CI	*P*-value	Aware of the Nagahama study (*n*=381) %	Know nothing (*n*=376) %	OR	95 % CI	*P*-value
Age group years)										
30–39	25.2	74.8	1			40.3	59.7	1		
40–49	20.9	79.1	0.78	0.43–1.41	0.415	43.7	56.3	1.15	0.74–1.78	0.527
50–59	26.8	73.2	1.09	0.63–1.88	0.767	57.9	42.1	2.04	1.33–3.12	0.001
60–69	38.6	61.4	1.86	1.14–3.05	0.013	55.9	44.1	1.89	1.25–2.84	0.003
Formal education period										
High school graduate and	30.4	69.6	1			47.9	52.1	1		
College and higher	31.1	68.9	0.97	0.69–1.35	0.844	53.9	46.1	0.79	0.59–1.06	0.109
Self-rated understanding of terminology										
High	37.3	62.7	1			62.8	37.2	1		
Middle	30.7	69.3	0.74	0.51–1.08	0.122	51.5	48.5	0.63	0.43–0.92	0.018
Low	21.1	78.9	0.45	0.29–0.69	<0.001	41.6	58.4	0.42	0.29–0.62	<0.001
Awareness of benefits										
Helpful for disease diagnosis	91.1	79.0	2.73	1.60–4.66	<0.001	90.2	77.0	2.76	1.80–4.23	<0.001
Helpful for disease treatment	93.2	78.3	3.77	2.10–6.78	<0.001	89.1	76.5	2.51	1.66–3.79	<0.001
Helpful for disease prevention	90.2	75.7	2.96	1.78–4.92	<0.001	89.1	77.0	2.44	1.61–3.70	<0.001
Concerns										
Financial infusion	79.7	77.7	1.13	0.75–1.69	0.572	78.7	74.4	1.27	0.90–1.80	0.180
Privacy concerns	57.7	61.7	0.85	0.61–1.19	0.339	56.3	52.8	1.15	0.86–1.55	0.355
Discriminations	40.4	45.0	0.83	0.59–1.16	0.275	38.5	37.2	1.06	0.78–1.44	0.711
Unexpected negative effects	34.0	42.8	0.68	0.49–0.97	0.034	30.5	32.6	0.91	0.66–1.25	0.564
Cloned human beings	33.5	37.0	0.86	0.61–1.22	0.387	29.0	32.0	0.87	0.63–1.20	0.391
Belief										
Company or government bodies use genome information	44.5	45.6	0.96	0.69–1.34	0.796	39.9	38.8	1.05	0.78–1.42	0.758

### Attitudes toward the use of genetic information for medical purposes

**Fig 1:**
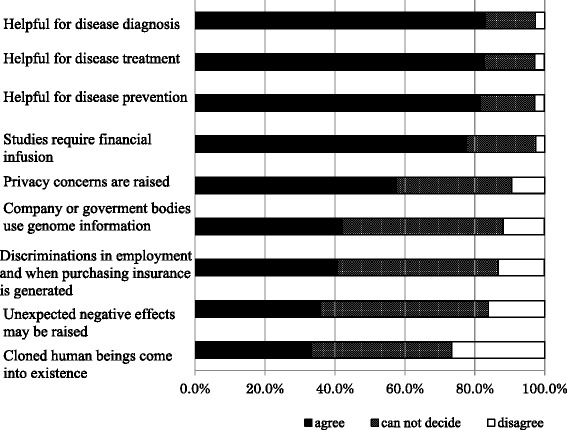
Attitudes toward the use of genetic information for medical purposes

More than 80 % of respondents agreed that the use of genetic information for medical purposes is “helpful for disease diagnosis,” “helpful for disease treatment,” and “helpful for disease prevention.” All three “awareness of benefit” items exhibited high mutual correlations (Cronbach’s alpha = 0.917). Less than half of the respondents agreed that “companies or government bodies use genome information,” “discrimination in employment and when purchasing insurance is generated,” “cloned human beings come into existence,” and “unexpected negative effects may be raised.” All five “concern” items also exhibited high mutual correlations (Cronbach’s alpha = 0.794) (Fig. 1). Those who were aware of the Nagahama study showed significantly higher awareness of the benefit of genomic studies than those who were unaware [diagnosis, *χ*^2^ (1) = 36.93 *p* < 0.001; treatment, *χ*^2^ (1) = 39.80, *p* < 0.001; prevention, *χ*^2^ (1) = 39.23, *p* < 0.001]. On the other hand, males who knew nothing showed significantly higher concerns that “unexpected negative effects may be raised” [*χ*^2^ (1) = 4.50, *p* = 0.034]. However, other significant differences were not found between those who were aware of the Nagahama study and those who knew nothing about the study.

The effects of awareness of the Nagahama study, concerns, the belief that companies or government bodies might use genome information, and the self-rated understanding of the terminology were also examined in relation to the “awareness of benefits.” According to the result, both males and females who were aware of the project’s contents, perceived high and middle level of understanding of the terminology and the concern item of “studies require financial infusion” significantly agreed with the three awareness of benefit items of “helpful for disease diagnosis,” “helpful for disease treatment,” and “helpful for disease prevention.” Particularly, in females, “cloned human beings come into existence” was positively and “unexpected negative effects may be raised” was negatively associated with the awareness of the benefit items (Table 3).

**Table 3:** Relationship between awareness of the benefit of genome study and awareness of the Nagahama study, self-rated understanding of terminology, concern, and belief of usage of genetic information in companies or government bodies from Logistic Regression Analysis

	Male (*n*=704)					Female (*n*=769)				
	The use of genetic information is helpful for disease treatment					The use of genetic information is helpful for disease treatment				
	Agree (*n*=554)	Neutral or Disagree	OR^a^	95 % CI	*P*-value	Agree (*n*=591)	Neutral or Disagree	OR^a^	95 % CI	*P*-value
	n (%)	n (%)				n (%)	n (%)			
High awareness of the Nagahama study	191 (34.8)	14 (12.4)	3.82	1.93–7.57		326 (55.6)	40 (33.3)	2.63	1.63–4.24	<0.001
Self-rated understanding of terminology										
High	231 (41.8)	19 (16.5)	1			168 (28.8)	15 (12.6)	1		
Middle	206 (37.3)	30 (26.1)	0.66	0.34–1.29	0.223	224 (38.4)	37 (31.1)	0.72	0.36–1.42	0.342
Low	116 (21.0)	66 (57.4)	0.24	0.12–0.46	<0.001	192 (32.9)	67 (56.3)	0.49	0.27–0.98	0.045
Concerns										
Financial infusion	456 (84.0)	60 (52.2)	4.37	2.49–7.69	<0.001	473 (81.4)	65 (53.7)	2.98	1.79–4.97	<0.001
Privacy concerns	343 (62.5)	56 (49.6)	0.72	0.39–1.32	0.284	335 (56.9)	53 (43.8)	1.00	0.57–1.74	0.993
Discriminations	250 (45.3)	39 (34.2)	1.38	0.74–2.58	0.315	234 (39.9)	38 (31.4)	0.77	0.43–1.38	0.386
Unexpected negative effects	228 (41.4)	38 (33.0)	1.02	0.54–1.93	0.963	192 (32.6)	33 (27.5)	0.49	0.26–0.90	0.022
Cloned human beings	203 (37.0)	35 (30.4)	0.87	0.46–1.66	0.682	191 (32.5)	24 (20.2)	2.27	1.16–4.46	0.017
Belief										
Company or government bodies use genome information	267 (48.8)	33 (29.7)	1.35	0.77–2.36	0.299	251 (42.7)	30 (25.0)	1.71	0.98–3.00	0.060

^**a**^Adjusted according to age and formal education duration

### Willingness to participate in drug susceptibility genetic testing

When asked whether they would be willing to undergo drug susceptibility genetic testing, 48.5 % of the respondents revealed positive attitudes, 29.7 % could not decide, and 21.7 % revealed negative attitudes (Table 1). Moreover, 55 % of those who agreed that the use of genetic information for medical purposes is “helpful for disease treatment” were willing to undergo drug susceptibility genetic testing. There was no significant difference between males and females.

Logistic regression analyses were performed to examine the association of the willingness to participate in drug susceptibility genetic testing to be informed of the drug’s effectiveness or adverse effects and attitudes toward genetic testing. According to the results presented in Table 4, males who were aware of the project’s contents, had a high level of self-rated understanding of the terminology, and had high awareness of benefit of genetic testing for disease treatment were more willing to donate their DNA for drug susceptibility genetic testing compared with the reference group of males who were unaware of the Nagahama study, perceived their understanding of terminology middle and low, and had a low awareness of the benefit of genetic testing for disease treatment. Females who had high and middle levels of self-rated understanding of the terminology and had high awareness of benefit of genetic testing for disease treatment were more willing to undergo drug susceptibility genetic testing than the reference group of females who perceived their understanding of terminology low and had a low awareness of the benefit of genetic testing. There was no association with positive attitude toward such testing and awareness of the project’s contents in females. Significant differences were not observed between the willingness to participate in drug susceptibility genetic testing and either concerns about genome studies or the belief of usage of genome information in companies or government bodies in both sexes.

**Table 4:** Relationship between the willingness toward drug susceptibility genetic studies and the attitudes toward genome studies from Logistic Regression Analysis

	Male (*n*=704 )					Female (*n*=769)				
	Willingness toward drug susceptibility genetic studies					Willingness toward drug susceptibility genetic studies				
	Yes (*n*=339)	Neutral or No (*n*=348)	OR^a^	95 % CI	*P*-value	Yes (*n*=360)	Neutral or No (*n*=391)	OR^a^	95 % CI	*P*-value
	n (%)	n (%)				n (%)	n (%)			
High awareness of the Nagahama study	131 (38.9)	77 (22.5)	2.06	1.40–3.03	<0.001	200 (56.0)	178 (46.4)	1.28	0.92–1.79	0.150
Self-rated understanding of terminology										
High	162 (47.9)	88 (25.3)	1			117 (32.8)	67 (17.5)	1		
Middle	120 (35.5)	124 (35.6)	0.58	0.39–0.87	0.008	148 (41.5)	119 (31.2)	0.81	0.53–1.22	0.307
Low	56 (16.6)	136 (39.1)	0.33	0.20–0.54	<0.001	92 (25.8)	196 (51.3)	0.35	0.22–0.55	<0.001
The use of genetic information is helpful for disease treatment	311 (94.0)	241 (71.9)	3.41	1.93–6.01	<0.001	317 (90.1)	272 (76.0)	2.51	1.54–4.07	<0.001
Concerns										
Financial infusion	268 (82.5)	247 (74.6)	0.98	0.60–1.60	0.941	280 (80.2)	260 (73.2)	0.90	0.59–1.38	0.629
Privacy concerns	199 (60.9)	197 (59.3)	0.93	0.59–1.45	0.739	200 (56.7)	190 (52.9)	0.94	0.63–1.40	0.762
Discriminations	139 (42.0)	147 (44.3)	0.83	0.53–1.28	0.289	145 (41.2)	127 (35.7)	1.17	0.78–1.75	0.459
Unexpected negative effects	131 (39.7)	133 (39.9)	1.09	0.70–1.70	0.696	117(33.1)	112 (31.1)	0.97	0.63–1.49	0.891
Cloned human beings	114 (34.5)	122 (36.9)	0.85	0.54–1.34	0.934	143 (40.7)	137 (38.3)	1.04	0.67–1.61	0.872
Belief										
Company or government	161 (49.4)	137 (41.4)	1.32	0.89–1.94	0.167	143 (40.7)	137 (38.3)	0.89	0.61–1.29	0.529

^**a**^Adjusted according to age and formal education duration

## Discussion

This article examined the effects of a community-based genomic–epidemiologic study on residents of Nagahama, Japan regarding their attitudes toward the project and genetic testing.

Respondents who were aware of the contents of the study perceived their understanding of terminology to be high. This was contrary to our expectation that the awareness of the study contents did not correlate with their self-rated understanding terminology as Nagahama City and Kyoto University try hard to keep every citizen informed about the Nagahama study by providing leaflets several times to all houses [14]. One reason might be that participants in the Nagahama study could enhance their understanding of terminology through the study’s briefing paper, and another reason might be that those who were interested in genome studies could also attend symposia or science cafés because science communication activities are mainly accepted by those who are already interested in science and have positive attitudes toward science, as Gottweis [28] suggested.

Previous studies have shown that the public generally has positive attitudes toward genetic studies [15–19]. Similarly, the present study found that more than 80 % of the respondents were aware of the benefits of genetic studies. Those who were aware of the project’s contents and perceived their comprehension of the terminology well were more aware of the benefits than those who had lower awareness. This result supports the idea that experiences and familiarity with genetic testing are associated with positive attitudes toward it. In a nationwide study in Japan [17], those who had learned about genetics in school and had heard of the term “genetic testing” approved of the promotion of genomic studies. In Dutch studies [16, 23], the public attitude changed over time even though their experience with genetic testing had not increased. More people endorsed the ideas that “genetic knowledge helps people live longer” and “genetic study should be promoted” in 2010 than in 2002. They suggested that the public do not perceive neonatal and prenatal screening tests as genetic tests. However, there is a chance that the public unconsciously glances over genetic issues in today’s society. Condit [12] suggested that people accept a new technology as the natural order when it becomes familiar over time. Most citizens usually see genome studies as being outside of the natural order. The Nagahama study program was perceived as an extensive and free health check-up program that ultimately inspired 10,084 residents to become involved [14]. In this case, because of the number of participants, this method may be effective to familiarize the general public with genetic studies and its outcomes. Alhakami & Slovic [29] suggested that people tended to judge the benefits as high when they felt the activity was favorable, which may apply in the case.

Lids et al. [30] clarified that research participants have a “personal” frame, while researchers have a “science” frame. Therapeutic misconception arises not from a lack of information but from a difference in cognitive frame. Therefore, they posit that scientific reframing of what is involved in a clinical trial is necessary. The Nagahama study has been promoted by the catchphrase “for your children and grandchildren” to avoid a “science” frame. One third of citizens live in their native community and one fourth of citizens live with their extended family consisting of more than three generations of family members [31]. Belief of the study’s benefits for their children and grandchildren may attract them to participate and become aware of the benefits of the genome study.

Almost half of the respondents had a positive attitude toward donating their DNA for drug susceptibility genetic testing, while approximately 30 % could not decide and roughly 20 % had a negative attitude. A logistic regression analysis was conducted to explore the factors associated with willingness to participate in drug susceptibility genetic testing. According to the results, both males and females who had positive attitudes toward such testing had high levels of self-rated understanding of the terminology and a high degree of awareness of the benefits of genetic studies.

Kobayashi and Satoh [32] used the Internet to explore Japanese attitudes toward pharmacogenomics research. Of their respondents, 45.3 % had a positive attitude and 46.3 % were neutral toward participating in pharmacogenomics research when taking medications. However, the willingness to participate increased to 61.7 % positive and decreased to 30.6 % neutral when experiencing severe adverse drug reactions. The proportion of respondents who refuse to participate was similar in both scenarios (8.3 % vs. 7.6 %, respectively) in their study. The reason for the disparity between Kobayashi’s study and ours could be the use of the Internet and a correspondingly higher interest in scientific issues compared with those in the present study.

Haga et al. [33] investigated the American public’s attitude toward pharmacogenomics research. Of their respondents, 92 % had positive attitudes toward testing to assist with drug selection and 73 % approved of testing to predict mild side effects. Respondents in their study were markedly more positive compared with respondents in our and Kobayashi’s studies [32]. Ishiyama et al. [17] assume that Japanese tend to be prudent in decision-making. Our results support them.

In addition, 78 % of respondents in the Haga et al. stated they were unwilling to undergo drug susceptibility genetic testing if their DNA or test results would be shared without their permission [33]. The authors suggest that the respondents might be unaware that federal law bans genetic discrimination by health insurers or employers, which may have effected their results. In the present study, the belief that companies or government bodies may use genetic information was not associated with willingness toward testing. Nagahama city government enacted an ordinance, the “Nagahama Rule,” to manage the Nagahama study. The mission was to prioritize citizens’ dignity and that might have encouraged citizens’ trust in the study.

Awareness of the project contents was associated with the willingness to participate in drug susceptibility genetic testing in males but not in females. According to a review by the Office of Science and Technology and the Wellcome Trust [34], the supporters of science tend to be self-confident, and they generally have trust in the government and higher authorities. Recruitment for the Nagahama study was achieved through various public information channels as well as word-of-mouth communications. Thus, males who were inactive in their respective community associations were less aware of the Nagahama study, whereas females were more aware because volunteering was a routine community activity for them [14]. Conversely, males who were active in their community associations generally trusted regulatory systems. This might explain why the positive attitudes toward drug susceptibility genetic testing associated with the awareness of Nagahama study were only seen in males. For females, just being aware of community-based genome studies did not change the willingness to participate in drug susceptibility genetic testing. Females may be able to bring themselves to donate DNA toward genetic testing if they feel familiar with the genetic terminology. Condit [12] suggests that people tend to perceive a certain category as the natural state of affairs when the given category is familiar to them. For example, people may agree to donate their DNA for drug susceptibility genetic testing without reluctance if their family members and if trusted confidants recommend such involvement. Moreover, as Lids et al. suggest, it is necessary to provide information appropriate to the public’s cognitive frame of personal needs for drug susceptibility genetic testing. As genome cohort study is coherently the project to promote public health through the development of medicine within a “science frame,” while “extensive and free health check-up,” “study for your children and grandchildren,” and “genetic testing for revealing of association between drug effectiveness or drug adverse reaction and genomic markers” are different things within a “personal frame.” Timely and consecutive communication is required because the results are obtained a long time after participants provide consent for the study.

The present study has several limitations. First, participants were dichotomized into respondents who were aware of the project’s contents and those who didn’t know about the study at all; however, this approach may have introduced bias. In fact, those who were aware of the project contents were generally involved in community activities, were health conscious, and had trust in the government. This self-selecting bias may have altered the results.

Second, this study itself may become a public relation tool for the Nagahama study, and consequently, influence respondents’ attitudes toward genetic research. However, we found that the attitude of subjects who were aware about the study contents before the study period was significantly different from that of subjects who did not know about the study content before the study period. This difference persisted even if subjects who were unaware of the contents gained knowledge during the study period.

Third, self-rated understanding of terminology may not indicate true comprehension. According to Ladwig et al. [24], perceived knowledge affects understanding in a different way than factual knowledge. Perceived knowledge could be influenced by specific heuristics, whereas factual knowledge could not. Hypothetically, perceived knowledge is more supportive of technology. Future studies should investigate the differences of influence between perceived and factual knowledge for public understanding of genome study. Finally, this study involved cooperation between the local government and a university, which is a rare scenario in Japan. Thus, the results of such collaboration may not apply to other communities.

It is necessary to conduct future research about the gap between the awareness of the benefits of genome study and the willingness to undergo drug susceptibility genetic testing. We hope to conduct a series of interviews to explore the reasons for negative attitudes toward drug susceptibility genetic testing, which might be a potential abhorrence, differences in context, the misconception as specific treatment for serious diseases but general diagnose, lack of information, or a matter of communication.

## Conclusions

This study examined how a community-based genome cohort study affects the attitudes of the general public toward such projects. In this case, the Nagahama study was perceived by the residents as a reasonable health check-up program, which eventually helped familiarize them with genetic studies and earn their intuitive trust. However, even though similar projects can promote greater awareness of the benefits of genetic studies, awareness may not always lead to greater willingness to participate in genome studies and clinical applications. In particular, females did not display willingness to participate in related activities, such as drug susceptibility genetic testing. This indicates that some citizens feel genome studies are unfamiliar and risky. Luhmann [35] stated that “Trust is based on a circular relation between risk and action,” and “Familiarity is an unavoidable fact of life; trust is a solution for specific problems of risk.” Genome studies, in which a subject or their acquaintances participated along with the possibility that the studies could improve the health of children and grandchildren, engender a perception of less risk and greater familiarity. Certainly, the Nagahama study may be understood as a free and extensive health check-up program to some degree. However, it would be probably safe to assume that familiarity leads to understanding and future action because trust does not work in an unfamiliar world.
