# Erratum to: Experiences and attitudes of residents regarding a community-based genome cohort study in Japan: a population-based, cross-sectional study

**DOI:** 10.1186/s12920-016-0238-x

**Published:** 2017-01-12

**Authors:** Keiko Miyamoto, Miho Iwakuma, Takeo Nakayama

**Affiliations:** 1 Department of Medical Communication, Kyoto University School of Public Health, Yoshidakonoe-cho, Sakyo-ku, Kyoto, #6068501 Japan; 2 Department of Health Infomatics, Kyoto University School of Public Health, Yoshidakonoe-cho, Sakyo-ku, Kyoto, #6068501 Japan

## Erratum

After the publication of this article the authors noticed that an incorrect version of Table 3 is shown. The correct version can be seen on the following page.

**Table 3:** Relationship between awareness of the benefit of genome study and awareness of the Nagahama study, self-rated understanding of terminology, concern, and belief of usage of genetic information in companies or government bodies from Logistic Regression Analysis

	Male (n = 704)					Female (n = 769)				
	The use of genetic information is helpful for disease treatment					The use of genetic information is helpful for disease treatment				
	Agree (n = 554)	NEW: Neutral or Disagree (n=115)	OR^a^	95%CI	P-value	Agree (n = 591)	NEW: Neutral or Disagree (n=121)	OR^a^	95%CI	P-value
	n (%)	n (%)				n (%)	n (%)			
High awareness of the Nagahama study	191 (34.8)	14 (12.4)	3.82	1.93–7.57	<0.001	326 (55.6)	40 (33.3)	2.63	1.63–4.24	<0.001
Self-rated understanding of terminology										
High	231 (41.8)	19 (16.5)	1			168 (28.8)	15 (12.6)	1		
Middle	206 (37.3)	30 (26.1)	0.66	0.34–1.29	0.223	224 (38.4)	37 (31.1)	0.72	0.36–1.42	0.342
Low	116 (21.0)	66 (57.4)	0.24	0.12–0.46	<0.001	192 (32.9)	67 (56.3)	0.49	0.27–0.98	0.045
Concerns										
Financial infusion	456 (84.0)	60 (52.2)	4.37	2.49–7.69	<0.001	473 (81.4)	65 (53.7)	2.98	1.79–4.97	<0.001
Privacy concerns	343 (62.5)	56 (49.6)	0.72	0.39–1.32	0.284	335 (56.9)	53 (43.8)	1.00	0.57–1.74	0.993
Discriminations	250 (45.3)	39 (34.2)	1.38	0.47–2.58	0.315	234 (39.9)	38 (31.4)	0.77	0.43–1.38	0.386
Unexpected negative effects	228 (41.4)	38 (33.0)	1.02	0.54–1.93	0.963	192 (32.6)	33 (27.5)	0.49	0.26–0.90	0.022
Cloned human beings	203 (37.0)	35 (30.4)	0.87	0.46–1.66	0.682	191 (32.5)	24 (20.2)	2.27	1.16–4.46	0.017
Belief										
Company or government bodies use genome information	267 (48.8)	33 (29.7)	1.35	0.77–2.36	0.299	251 (42.7)	30 (25.0)	1.71	0.98–3.00	0.060

^a^Adjusted according to age and formal education duration
